# HMGA2 facilitates colorectal cancer progression via STAT3-mediated tumor-associated macrophage recruitment

**DOI:** 10.7150/thno.65411

**Published:** 2022-01-01

**Authors:** Xin Wang, Jian Wang, Jiahui Zhao, Hao Wang, Jing Chen, Jingjing Wu

**Affiliations:** 1 Department of Pathology & Pathophysiology, and Department of Colorectal Surgery of the Second Affiliated Hospital, Zhejiang University School of Medicine, Hangzhou, Zhejiang, China.; 2 Key Laboratory of Disease Proteomics of Zhejiang Province, Key Laboratory of Cancer Prevention and Intervention of China National Ministry of Education, Zhejiang University School of Medicine, Hangzhou, China.; 3 Department of Colorectal Surgery and Oncology, the Second Affiliated Hospital, Zhejiang University School of Medicine, Hangzhou, China.

**Keywords:** colorectal cancer, HMGA2, STAT3, macrophages

## Abstract

**Rationale:** Tumor-associated macrophages (TAMs), generally displaying the pro-tumor M2-like phenotype, strongly influence the progression of colorectal cancer (CRC) via their immunosuppressive activities. The high-mobility gene group A2 (HMGA2), an oncoprotein, is aberrantly overexpressed in CRC cells. However, the mechanisms by which tumor-derived HMGA2 modulates tumor microenvironment in CRC remain poorly understood.

**Methods:** *In vivo* subcutaneous tumor xenograft model, azoxymethane (AOM)/dextran sodium sulfate (DSS)-induced tumor mouse model and *in vitro* co-culture assays were used to investigate the Hmga2 role in TAM recruitment and polarization. Luciferase and chromatin immunoprecipitation (ChIP) assays were applied to examine the mechanism of HMGA2-mediated transcriptional regulation of signal transducer and activator of transcription 3 (STAT3). The CD68 correlation with patient outcome was analyzed in 167 human CRC tissues.

**Results:** We found that HMGA2 in cancer cells promoted macrophage recruitment and M2 polarization *in vitro* and *in vivo*. HMGA2 directly bound to the *STAT3* promoter to activate its transcription and subsequently induced CCL2 secretion, thus promoting macrophage recruitment. Our results from human CRC specimens also revealed a strong positive association between HMGA2 expression in tumor cells and CD68 expression in the stroma. We further showed that patients with an elevated CD68 expression had an unfavorable overall survival in all of the patients or in the subgroup with negative distant metastasis.

**Conclusion:** Our work uncovers new insight into the link between the HMGA2/STAT3/CCL2 axis and macrophage recruitment in CRC. These findings provide a novel therapeutic option for targeting the HMGA2/STAT3/CCL2 axis in CRC.

## Introduction

Colorectal cancer (CRC) is a major public health issue due to its high incidence and death rates globally 1. Apart from environmental factors, such as smoking, alcohol, and obesity, genetic and epigenetic alterations contribute to the development and progression of CRC, including loss-of-function mutations of p53 and APC and gain-of-function of β-catenin and MAPK 2. Although significant improvement in overall survival has been achieved over the past few decades, the molecular mechanisms underlying the pathogenesis of CRC are still unclear. Thus, the need to better understand the underlying biological processes and develop better treatment approaches is crucial.

High mobility group AT-hook 2 (HMGA2) is a group of small chromatin-associated proteins that show rapid electrophoretic mobility patterns in polyacrylamide gels 3. It acts as an architectural transcription factor that directly binds to DNA sequences, thus altering the structure of DNA and modulating the transcription of target genes 3, 4. Our previous studies showed that HMGA2 exhibited diverse biological functions, contributing to CRC progression such as promoting CRC metastasis by directly activating the transcription of *FN1* and *IL11* 5, and enhancing MDM2-mediated p53 ubiquitination and degradation 6. In our previous study, we reported that elevated HMGA2 level was correlated with poor survival in CRC patients 5. Similar findings were observed by Wang *et al.* 7 However, there are limited studies elucidating the influence of HMGA2 in regulating the CRC tumor microenvironment (TME).

Recently, TME has attracted increasing attention and has been shown to play a vital role in tumor initiation and progression. TME is complex and heterogeneous consisting of innate and adaptive immune cells, fibroblasts, endothelial cells, and extracellular matrix 8. Various immune suppressive cells are considered to create a tolerant microenvironment, including tumor-associated macrophages (TAMs), regulatory T cells (Treg), regulatory B cells (Breg), and myeloid-derived suppressor cells (MDSCs) 9, 10. Macrophages are divided into two phenotypes: the pro-inflammatory M1 type and the anti-inflammatory M2 type 11, 12. M1 macrophages produce pro-inflammatory molecules that trigger inflammation and execute anti-tumor effects, such as TNF-α, IL-12, IL-23, and iNOS. In contrast, M2 macrophages generate anti-inflammatory cytokines that contribute to exert immunosuppressive and pro-tumor activities, such as TGF-β, IL-10, and Arg-1 13. TAMs generally exhibit M2-like properties and their role in driving CRC pathogenesis needs to be clarified. Therefore, elucidation of the crosstalk between cancer cells and TAMs is important for understanding the underlying mechanism and developing novel therapeutic approaches targeting TME.

In this study, we found that overexpression of HMGA2 in cancer cells promoted macrophage recruitment and M2 polarization in TME by upregulating signal transducer and activator of transcription 3 (STAT3)-mediated CCL2 secretion in CRC, as evidenced by *in vivo* and *in vitro* experiments. Our data further showed that HMGA2 directly activated *STAT3* transcription and the *STAT3* promoter regions -743/-730 and -585/-576 were essential for HMGA2-mediated *STAT3* promoter activity. A strong positive association between HMGA2 and CD68 expression was also observed in human CRC specimens. The tumors with high CD68 had a shorter overall survival rate in all of the patients and in the subgroup with negative distant metastasis. Our study revealed an important role of the HMGA2/STAT3/CCL2 axis in facilitating TAM recruitment in CRC and suggested its potential as a target for therapeutic intervention in CRC.

## Materials and Methods

### Patients and samples

This study used 167 CRC tissues obtained from the Second Affiliated Hospital of Zhejiang University School of Medicine between October 2009 and December 2012. The clinicopathologic characteristics of patients were summarized in Table S1. Tissue microarrays (TMAs) were constructed from paraffin-embedded blocks. The study was approved by the Ethics Committee of the Zhejiang University School of Medicine.

### Mice

All animal studies were carried out in compliance with the National Institute of Health Guide for the Care and Use of Laboratory Animals and approved by the Ethics Committee of the Zhejiang University School of Medicine. BALB/c and C57BL/6 female mice, aged 4-6 weeks, were purchased from Shanghai SLAC Laboratory Animal Co., Ltd. (Shanghai, China). All mice were maintained in a pathogen-free (SPF) environment in the Zhejiang University Laboratory Animal Center.

### RNA extraction and quantitative reverse transcriptase-polymerase chain reaction (RT-qPCR) analysis

Total RNA was separated from cells and samples using TRIZOL reagent (Invitrogen, Grand Island, NY, USA), and total RNA was reverse-transcribed to obtain cDNA by applying the PrimeScript RT reagent kit (TaKaRa). Then we carried out quantitative PCR using SYBR Green PCR Master Mix (Thermo Fisher) according to the manufacturer's protocol. All reactions were performed in triplicate. GAPDH was used as an internal control. The primer sequences are presented in Table S2.

### Luciferase assay

Serial deletion regions of the human STAT3 promoter (-139/+133, -1555/-140, -850/-140, -1555/-851 and -1555/+133) and the human HMGA2 promoter (-1365/+140) were cloned into the pGL3 vector (Promega). Mutations of the HMGA2 binding site in the STAT3 promoter (Mut 1, Mut 2 and Mut 3) were generated through site-directed mutagenesis (Stratagene). The primers for plasmid constructions were summarized in Table S3. Next, co-transfections of pGL3 (WT or Mut), pcDNA3.1 (NC or HMGA2) and pRL-TK were performed in HEK293T cells. After 48 hours, the cells were lysed and processed for the detection of the luciferase activities using the Dual-Luciferase Reporter Assay System (Promega). All experiments were carried out in triplicate.

### Chromatin immunoprecipitation (ChIP) assay

After cells were fixed in 1% formaldehyde in PBS for 10 minutes, they were lysed and sonicated to achieve chromatin fragmentation. Then, the lysates were immunoprecipitated with HMGA2 antibodies or negative control IgG (Cell Signaling Technology). The enrichment of HMGA2 protein with specific DNA fragments of STAT3 promoter was measured by PCR. Primers for ChIP-PCR amplification were presented in Table S4. Input chromatin collected without immunoprecipitation was used as the positive control.

### Statistical analysis

The data were depicted as mean ± standard deviation (SD). Comparisons between groups were analyzed using Student's t-tests. Kaplan-Meier analysis was applied to analyze overall survival. All statistical analyses were performed using SPSS 17.0 software (Chicago, IL, USA) or GraphPad Prism 7.0 software (San Diego, CA, USA). *P* < 0.05 was considered to be statistically significant.

## Results

### Hmga2 knockout in CRC cells suppressed TAM infiltration, M2 polarization, and CCL2 secretion in subcutaneous tumor models

**Figure 1:**
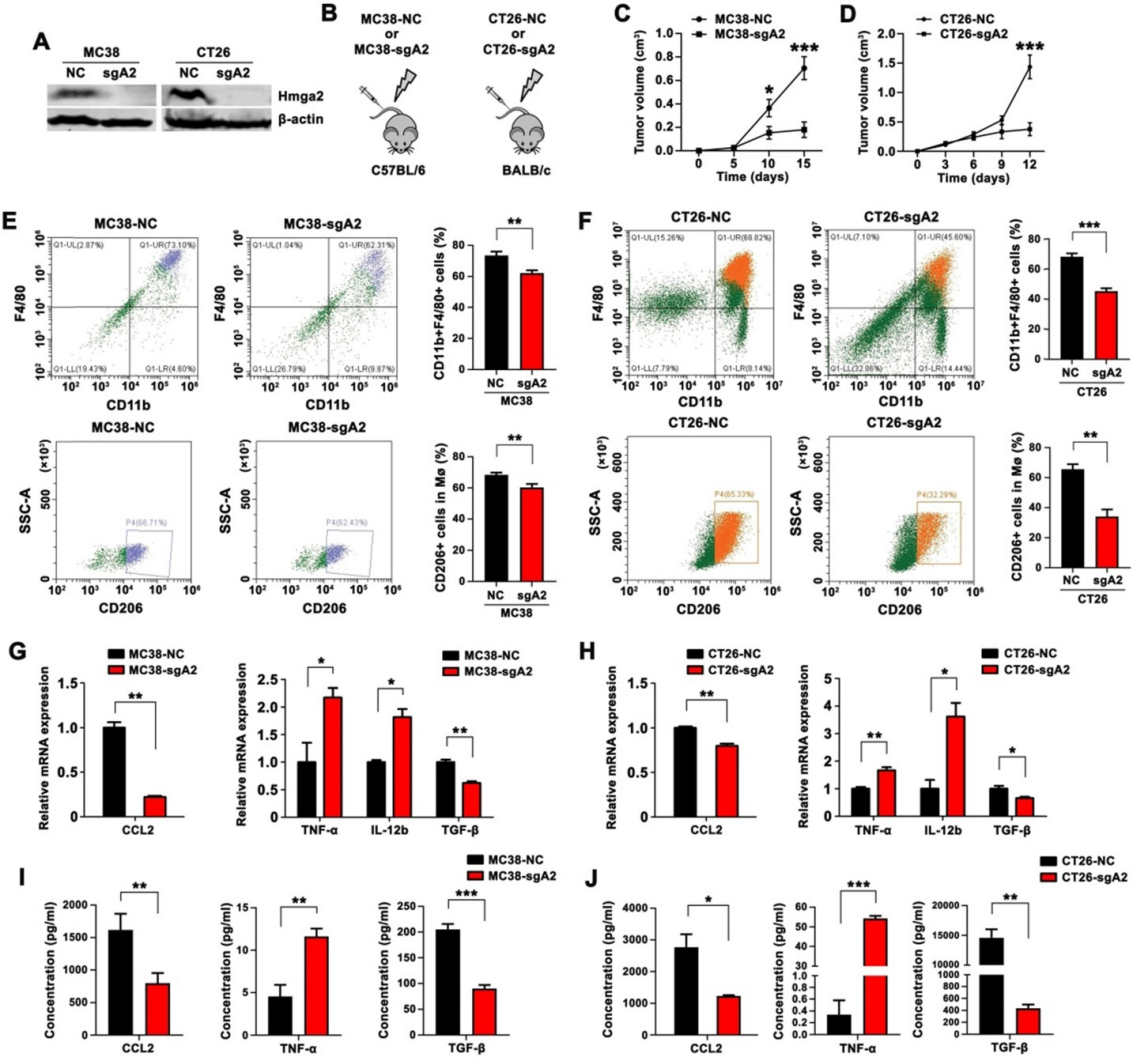
Knockout of Hmga2 in CRC cells suppressed TAM infiltration, M2 polarization, and CCL2 secretion in subcutaneous tumor models. A, Western blot analysis of Hmga2 levels in sgRNA-mediated Hmga2 knockout MC38 and CT26 cells. B, Schematic overview of subcutaneous tumor xenograft models. C-D, Growth curves of MC38-NC/MC38-sgA2 xenograft tumors in C57BL/6 mice (C), and CT26-NC/CT26-sgA2 xenograft tumors in BALB/c mice (D). E-F, Representative flow cytometry plots (left panel) and percentages (right panel) of CD11b^+^F4/80^+^ macrophages (upper panel) and CD11b^+^F4/80^+^CD206^+^ M2 macrophages (bottom panel) in tissues of MC38-NC/MC38-sgA2 xenograft tumors (E), and CT26-NC/CT26-sgA2 xenograft tumors (F). G-H, Quantitative RT-PCR analysis of CCL2, TNF-α, IL-12b, and TGF-β in MC38-NC/MC38-sgA2 xenograft tumor tissues (G), and CT26-NC/CT26-sgA2 xenograft tumors (H). I-J, ELISA analysis of CCL2, TNF-α and TGF-β concentration in the cultured supernatants from MC38-NC/MC38-sgA2 xenograft tumors (I), and CT26-NC/CT26-sgA2 xenograft tumors (J). Error bars indicate SD. *P < 0.05; **P < 0.01; ***P < 0.001.

Using the CRISPR/Cas9 technology, we generated stable Hmga2 knockout (Hmga2-KO) cells in the mouse CRC cell lines (MC38 and CT26) with specific sgRNA. The efficiency of Hmga2 knockout was verified by Western blot analysis (Figure 1A). C57BL/6 mice were subcutaneously inoculated with either the scrambled control or Hmga2-KO MC38 cells (MC38-NC and MC38-sgA2, respectively), while BALB/c mice were subcutaneously inoculated with CT26-NC or CT26-sgA2 cells (Figure 1B). The results showed that sgRNA-mediated knockout of Hmga2 significantly impaired tumor growth in both C57BL/6 and BALB/c subcutaneous tumor models (Figure 1C-D).

M2 macrophages are known to facilitate tumor growth and progression. To elucidate the role of Hmga2 in TAM recruitment and polarization, we employed flow cytometry to quantify the percentage of macrophages (CD11b^+^F4/80^+^) and M2 macrophages (CD11b^+^F4/80^+^CD206^+^). Mice bearing Hmga2 knockout MC38 tumors showed decreased infiltrating CD11b^+^F4/80^+^ macrophages and CD11b^+^F4/80^+^CD206^+^ M2 macrophages (Figure 1E), indicating that anti-tumor effects of Hmga2 depletion might involve mechanisms mediated by the recruitment and polarization of TAMs in CRC. Consistently, similar results were observed in mice bearing Hmga2 knockout CT26 tumors (Figure 1F). We also performed immunohistochemical analysis of F4/80 and CD206 in subcutaneous tumors. As shown in Figure S1A-B, MC38-sgA2 and CT26-sgA2 showed decreased immunoreactivities for F4/80 and CD206 compared with MC38-NC and CT26-NC tumors. Together, these results demonstrated that Hmga2 facilitated TAM recruitment and M2 polarization *in vivo*.

CCL2 is a crucial chemokine that contributes to macrophage recruitment and infiltration 14. To explore whether CCL2 was regulated by Hmga2, we performed qPCR to evaluate CCL2 expression and employed ELISA to analyze CCL2 secretion. As shown in Figures 1G and 1I, compared with the control group, the tumors from MC38-sgA2 cells displayed decreased CCL2 expression and produced less CCL2. Similarly, our results also confirmed that depletion of Hmga2 suppressed the expression and secretion of CCL2 in the CT26 subcutaneous tumor model by qPCR (Figure 1H) and ELISA assays (Figure 1J).

Next, to explore the association between Hmga2 depletion in CRC cells and macrophage polarization in TME, we evaluated the expressions of M1-relevant (TNF-α and IL-12b) and M2-relevant (TGF-β) cytokines. As shown in Figure 1G-H, qPCR results showed that Hmga2 knockout in CRC cells increased the expression of TNF-α and IL-12b in both MC38 and CT26 xenografts, while the TGF-β level was significantly reduced. The increased TNF-α and decreased TGF-β secretion in Hmga2-KO tumor tissues was further confirmed by ELISA in both MC38 and CT26 subcutaneous tumor models (Figure 1I-J). Collectively, our results demonstrated that Hmga2 knockout in CRC cells inhibited TAM infiltration, M2 polarization, and CCL2 secretion in subcutaneous tumor models.

### Intestinal epithelial-specific KI of Hmga2 promoted TAM infiltration, M2 polarization, and CCL2 secretion in AOM/DSS model

**Figure 2:**
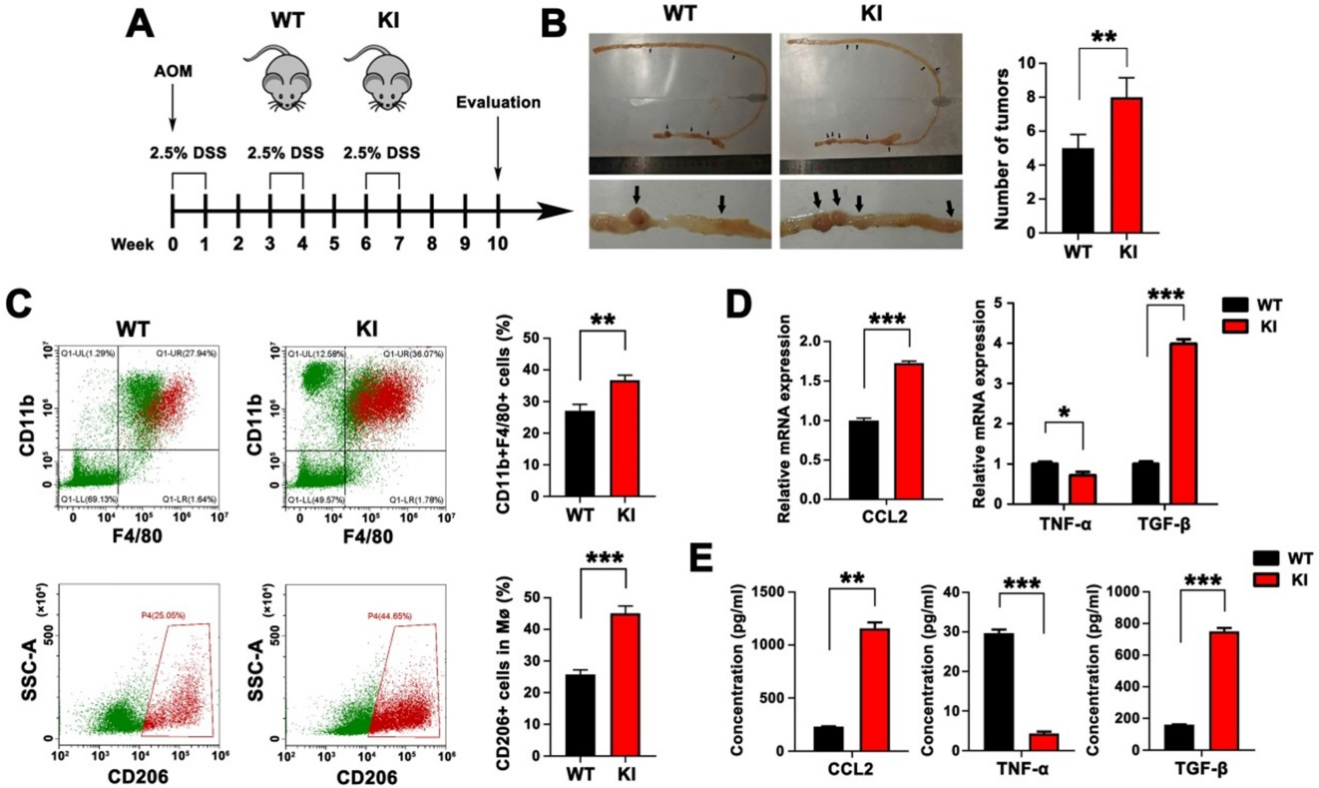
Intestinal epithelial-specific KI of Hmga2 promoted TAM infiltration, M2 polarization, and CCL2 secretion in the AOM/DSS model. A, Schematic overview of AOM/DSS mouse model in WT and Hmga2 KI mice. B, Representative images (left panel) and the total number (right panel) of intestinal tumors induced by AOM/DSS. C, Representative flow cytometry plots (left panel) and percentages (right panel) of CD11b^+^F4/80^+^ macrophages (upper panel) and CD11b^+^F4/80^+^CD206^+^ M2 macrophages (bottom panel) in intestinal tissues. D, Quantitative RT-PCR analysis of CCL2, TNF-α, and TGF-β in intestinal tissues. E, ELISA analysis of the concentrations of CCL2, TNF-α, and TGF-β in the cultured supernatants from intestinal tissues. Error bars indicate SD. *P < 0.05; **P < 0.01; ***P < 0.001.

We next sought to understand the involvement of intestinal epithelial-specific knock-in (KI) of Hmga2 in TAM infiltration and M2 polarization during CRC tumorigenesis. We used WT and intestinal epithelial-specific Hmga2 KI mice that were subsequently treated with AOM and DSS to induce colorectal tumors (Figure 2A). As shown in Figure 2B, Hmga2 KI mice developed more tumors in the intestine than WT mice. Intestinal tissues were collected and populations of macrophages (CD11b^+^F4/80^+^) and M2 macrophages (CD11b^+^F4/80^+^CD206^+^) were determined by flow cytometry. Compared with WT mice, increased percentages of CD11b^+^F4/80^+^ macrophages and CD11b^+^F4/80^+^CD206^+^ M2 macrophages were observed in intestinal tissues from Hmga2 KI mice following AOM/DSS administration (Figure 2C).

Furthermore, the level of chemokine CCL2 that mediates infiltration of macrophages was measured by qPCR and ELISA (Figure 2D-E). We found that Hmga2 KI caused a significant upregulation of CCL2 production in the intestinal tissues of mice. Also, qPCR results revealed that the expression of M1 cytokine (TNF-α) was significantly reduced in intestinal tissues from Hmga2 KI mice after AOM/DSS treatment, while M2 cytokine (TGF-β) was remarkably induced (Figure 2D). These findings were confirmed by ELISA (Figure 2E). In summary, these data suggested that intestinal epithelial-specific Hmga2 KI modulated the TME through facilitating TAM infiltration, M2 polarization and CCL2 secretion.

### HMGA2 promoted macrophage recruitment, M2 polarization, and CCL2 secretion *in vitro*

**Figure 3:**
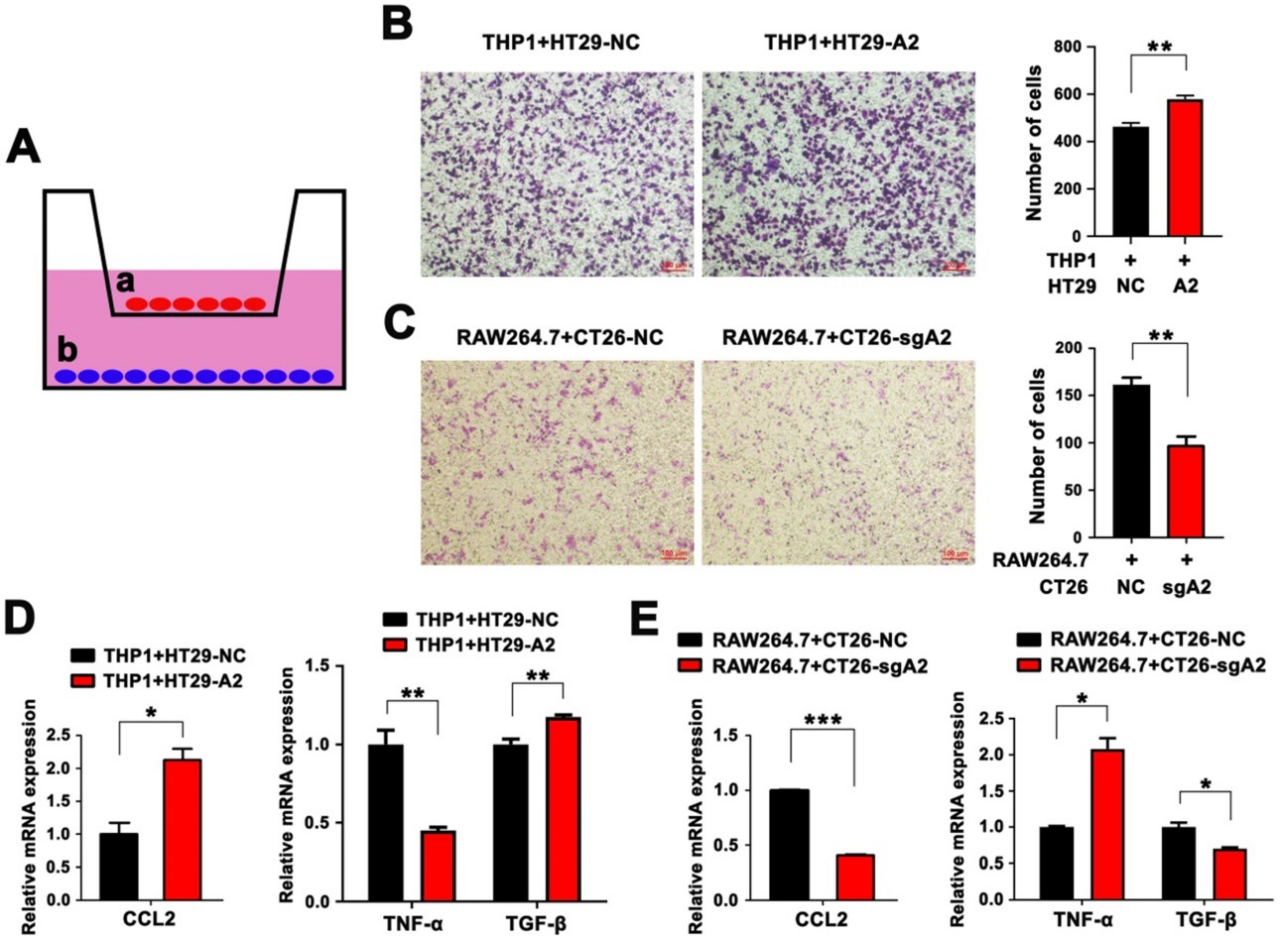
HMGA2 promoted macrophage recruitment, M2 polarization, and CCL2 secretion *in vitro*. A, Schematic overview of the *in vitro* co-culture model. B, Representative images (left panel) and quantification (right panel) of THP1 cells co-cultured with HT29-NC or HT29-A2 cells. C, Representative images (left panel) and quantification (right panel) of RAW264.7 cells co-cultured with CT26-NC or CT26-sgA2 cells. D, Quantitative RT-PCR analysis of CCL2 in HT29 cells (left panel), and TNFα and TGF-β in THP1 cells (right panel). E, Quantitative RT-PCR analysis of CCL2 in CT26 cells (left panel), and TNFα and TGF-β in RAW264.7 cells (right panel). Error bars indicate SD. *P < 0.05; **P < 0.01; ***P < 0.001.

To further illustrate whether HMGA2 contributes to TAM recruitment *in vitro*, we utilized a Transwell co-culture system of seeding macrophages in the upper compartment and CRC cells in the bottom chamber. As illustrated in Figure 3A, PMA-differentiated THP1 human monocytes were co-cultured with HT29 human colorectal cancer cells with or without HMGA2 overexpression (HT29-NC and HT29-A2, respectively), while RAW264.7 cells were co-cultured with CT26 murine colorectal cancer cells with or without Hmga2 KO (CT26-NC and CT26-sgA2, respectively). Interestingly, THP1 co-cultured with HT29-A2 displayed higher migratory ability than HT29-NC cells (Figure 3B). Conversely, the migration of RAW264.7 was markedly attenuated when co-cultured with CT26-sgA2 cells (Figure 3C). Subsequently, we investigated the CCL2 level in CRC cells by qPCR, showing that HMGA2 overexpression upregulated CCL2 expression in HT29 cells (Figure 3D), while Hmga2 KO downregulated its expression in CT26 cells (Figure 3E). In addition, TNF-α expression was decreased in THP1 cells after culturing with the conditioned medium from HT29-A2, whereas TGF-β showed the opposite trend (Figure 3D). Consistently, compared with the control group, increased TNF-α and decreased TGF-β levels were observed in RAW264.7 cells following treatment with the conditioned medium from CT26-sgA2 (Figure 3E). These studies further confirmed that HMGA2 promoted macrophage recruitment, M2 polarization, and CCL2 secretion *in vitro*.

### HMGA2 directly activated STAT3 transcription

**Figure 4:**
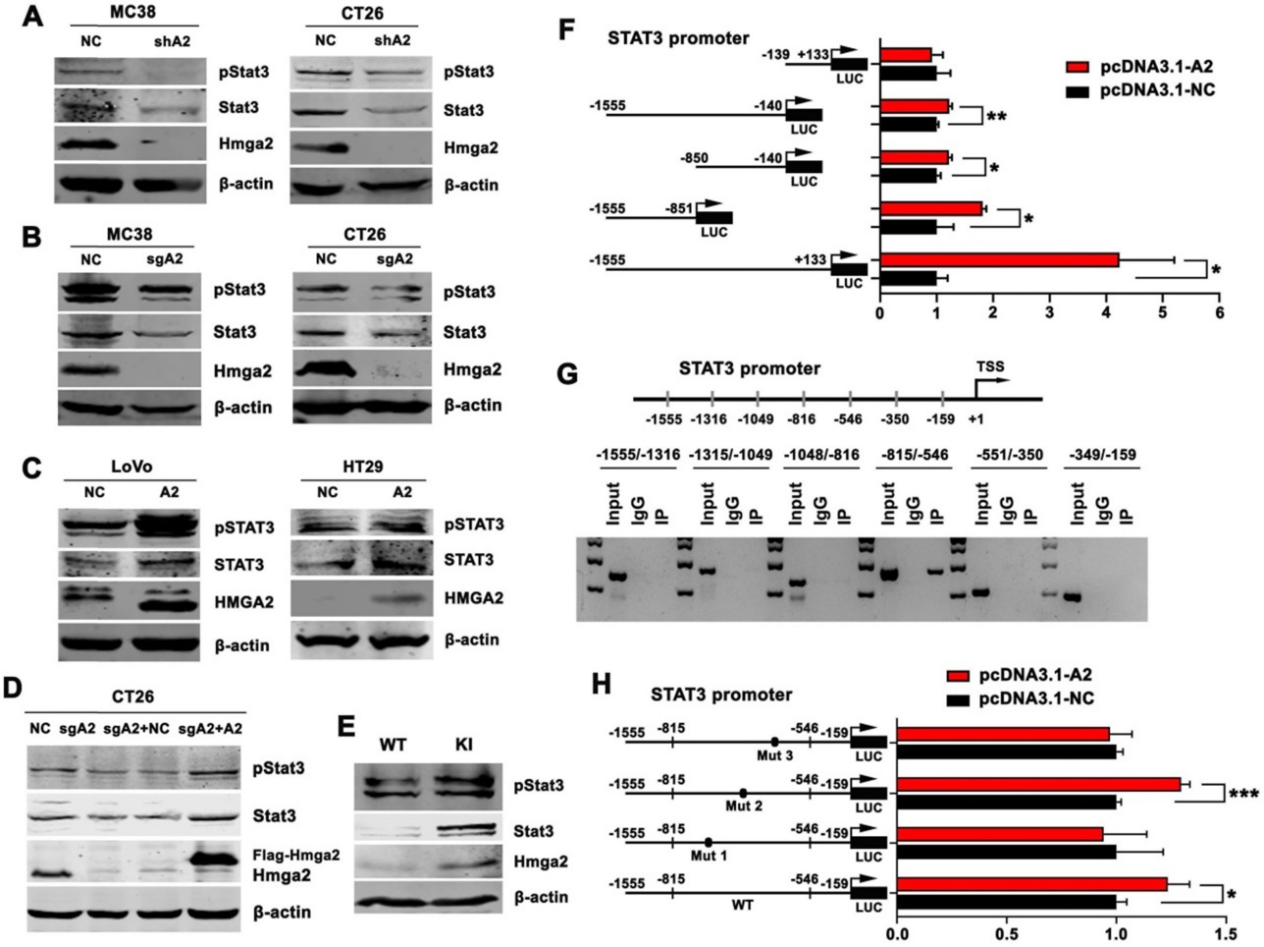
HMGA2 directly activated STAT3 transcription. A-E, Western blot analysis of pSTAT3^Tyr705^ and STAT3 protein levels in the indicated cells (A-D), and intestinal tissues of WT and KI mice (E). β-actin was used as an internal control. F, Luciferase activity of full-length or truncated STAT3 promoter constructs when co-transfected with control or HMGA2-overexpressing plasmids in HEK293T cells. G, ChIP analysis of HMGA2 enrichment at the indicated regions of STAT3 promoter. H, Luciferase activity of STAT3 promoter constructs containing WT or mutated sites (Mut 1, 2, and 3) when co-transfected with control or HMGA2-overexpressing plasmids in HEK293T cells. Error bars indicate SD. *P < 0.05; **P < 0.01; ***P < 0.001.

Previous studies have demonstrated that STAT3 has a key role in tumor immune tolerance 15. To investigate whether HMGA2 regulated immunosuppression of TAMs in the TME through the STAT3-dependent mechanism, we analyzed the relationship between HMGA2 and STAT3 in CRC. As presented in Figure 4A, shRNA-mediated knock-down of Hmga2 resulted in decreased levels of total and phosphorylated Stat3 (pStat3^Tyr705^) proteins in MC38 and CT26 cells. Consistently, sgRNA-mediated knock-out of Hmga2 significantly suppressed Stat3 and pStat3^Tyr705^ expression in MC38 and CT26 cells as seen by Western blotting (Figure 4B). Conversely, upregulation of STAT3 and pSTAT3^Tyr705^ was observed in HMGA2-overexpressing LoVo and HT29 cells compared to scrambled controls (Figure 4C). In addition, we transfected control or Hmga2 overexpression constructs into control or Hmga2-deficient CT26 cells (CT26-NC, CT26-sgA2, CT26-sgA2+NC, and CT26-sgA2+A2). As shown in Figure 4D, the reduced expression of Stat3 and pStat3^Tyr705^ by Hmga2 knockout was reversed when Hmga2 overexpression was restored. To better understand the regulatory mechanism between Hmga2 and Stat3 *in vivo*, we examined the Stat3, pStat3^Tyr705^, and Hmga2 expression in intestinal tissues of WT and KI mice by Western blotting. As shown in Figure 4E, we found that knock-in of Hmga2 enhanced Stat3 and pStat3^Tyr705^ levels.

To determine whether HMGA2 could activate STAT3 at the transcriptional level, we performed luciferase and ChIP assays. As illustrated in Figure 4F, we cloned five fragments of human *STAT3* promoter into the pGL3 vector, including -139/+133, -1555/-140, -850/-140, -1555/-851, and -1555/+133. The results showed that HMGA2 overexpression significantly stimulated luciferase activities of *STAT3* promoter regions -1555/-140, -850/-140, -1555/-851, and -1555/+133, indicating that the fragment -1555/-140 might contribute to the regulation of *STAT3* transcription by HMGA2 (Figure 4F). To further confirm it, we applied ChIP assay to verify the direct regulatory mechanism and identify the location of binding sites. As shown in Figure 4G, it showed that HMGA2 directly bound to the *STAT3* promoter and the HMGA2-binding sites were mainly located in the *STAT3* promoter region between -815 and -546.

Next, we mutated three HMGA2-binding sites flanking the promoter segments of *STAT3* (-815/-546) individually. The results displayed in Figure 4H demonstrated that induction of luciferase activities was significantly attenuated by transfection of constructs harboring mutation 1 (-743/-730, from TAATTACTCTATTT to TAGCCACTCTACGT) and 3 (-585/-576, from TATCTAACTA to TCTCGCGCTA), but not mutation 2 (-657/-644, from ATGTTCTTTTTGTT to ATGTCCTCGGTGTC). These observations suggested that HMGA2 enhanced *STAT3* transcription by binding directly to the -743/-730 and -585/-576 promoter regions of *STAT3*.

STAT3 is also known to be a transcriptional regulator that mediates the expression of inflammatory factors. We, therefore, explored the possibility that STAT3 regulated HMGA2 expression at the transcriptional level, thereby forming a feed-forward loop between HMGA2 and STAT3. However, as shown in Figure S2A-B, Western blotting and qPCR analysis showed that siRNA-mediated knockdown of Stat3 did not induce any significant change in the Hmga2 expression in MC38 and CT26 cells. Similarly, the luciferase activity from a construct harboring the HMGA2 promoter was unaffected by STAT3 overexpression (Figure S2C). These results demonstrated that STAT3 did not transcriptionally regulate HMGA2 expression in CRC. These findings suggested that HMGA2 directly activated STAT3 transcription, but STAT3 did not regulate HMGA2 transcription.

### Enhanced CCL2 expression and increased macrophage migration by HMGA2 overexpression in CRC cells depended on STAT3

**Figure 5:**
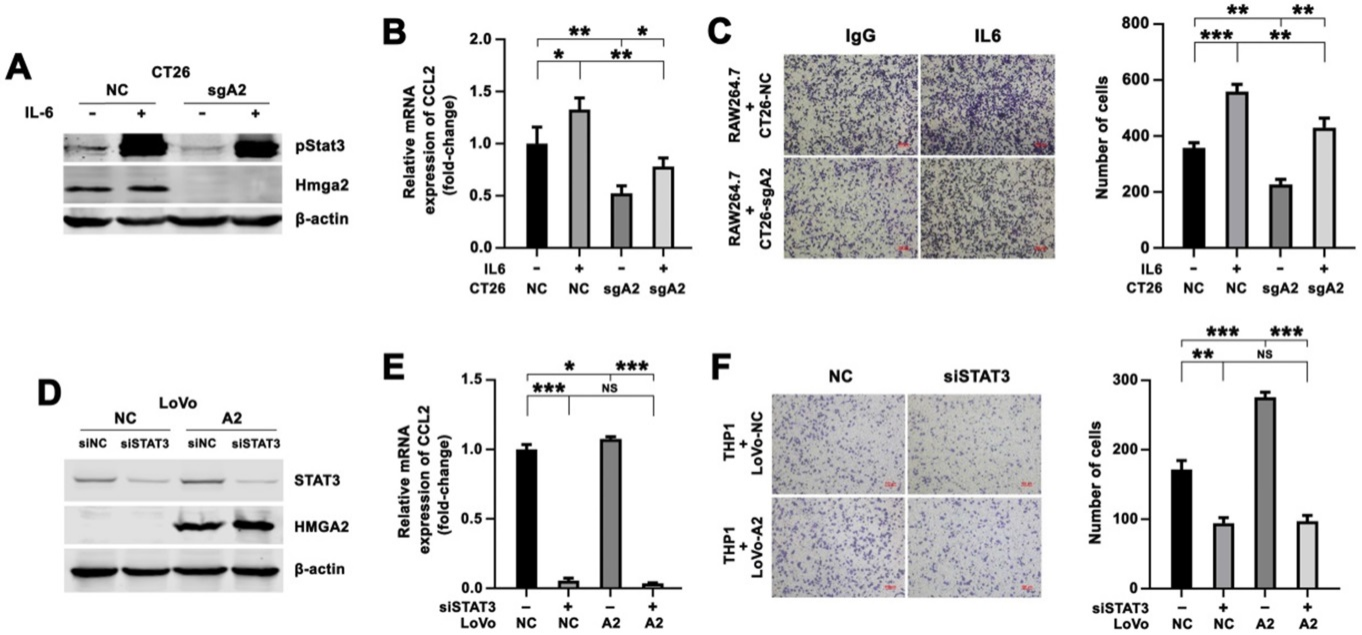
Enhanced expression of CCL2 and increased migration of macrophages by HMGA2 overexpression in CRC cells depended on STAT3. A-B, Western blot analysis of pStat3^Tyr705^ and Hmga2 (A), and quantitative RT-PCR analysis of CCL2 (B) in CT26-NC and CT26-sgA2 cells treated with control or recombinant murine IL6 protein. C, Representative images (left panel) and quantification (right panel) of RAW264.7 cells co-cultured with indicated CT26 cells treated with control or IL6. D-E, Western blot analysis of STAT3 and HMGA2 (D), and quantitative RT-PCR analysis of CCL2 (E) in LoVo-NC and LoVo-A2 cells transfected with control or siRNAs targeting STAT3. F, Representative images (left panel) and quantification (right panel) of THP1 cells co-cultured with indicated LoVo cells transfected with control or siRNAs targeting STAT3. Error bars indicate SD. *P < 0.05; **P < 0.01; ***P < 0.001; NS, not significant.

We determined whether Hmga2 regulated CCL2 expression in a Stat3-dependent manner by treating CT26-NC and CT26-sgA2 cells with recombinant murine IL6 to stimulate Stat3 and then co-culturing with RAW264.7 cells. IL6 treatment led to increased expression of pStat3^Tyr705^ (Figure 5A), strong upregulation of CCL2 expression in CRC cells (Figure 5B), and enhanced migration of RAW264.7 cells (Figure 5C).

Besides, siRNAs targeting STAT3 were introduced into LoVo-NC and LoVo-A2 cells, and the efficacy of STAT3 inhibition was evaluated by Western blotting (Figure 5D). We observed that the expression of CCL2 in the siSTAT3 group was lower than in the control group in LoVo-NC cells, indicating that STAT3 upregulated CCL2 expression (Figure 5E). We also found that HMGA2 overexpression resulted in increased expression of CCL2, but induction of CCL2 was abrogated after introducing siRNA targeting STAT3 (Figure 5E). A consistent result was observed in the Transwell co-culture system. STAT3 silencing in LoVo cells resulted in decreased migration of THP1 cells (Figure 5F). THP1 cells co-cultured with LoVo-A2 cells exhibited increased migratory ability, as compared to co-culture with LoVo-NC cells, but this increased migration was abrogated by introducing STAT3 siRNAs (Figure 5F).

In addition, to detect whether Hmga2 facilitated TAM infiltration via a STAT3-dependent way *in vivo*, we conducted rescue experiments in mice using the STAT3 inhibitor Stattic. Hmga2-knockout CT26 cells transfected with or without Hmga2 overexpression (CT26-sgA2+NC and CT26-sgA2+A2) were subcutaneously implanted into BALB/c mice, and then intraperitoneally treated with DMSO or Stattic. As shown in Figure S3A, Hmga2 overexpression increased the staining intensity of CD206, whereas Stattic treatment decreased it. However, increased TAM infiltration by Hmga2 overexpression was abrogated by Stattic treatment, suggesting that enhanced TAM infiltration by HMGA2 depended on STAT3 *in vivo*. These results demonstrated that HMGA2 upregulated CCL2 expression in CRC cells and promoted the migration of macrophages in a STAT3-dependent manner.

### Increased migration of macrophages by HMGA2 overexpression in CRC cells depended on CCL2

**Figure 6:**
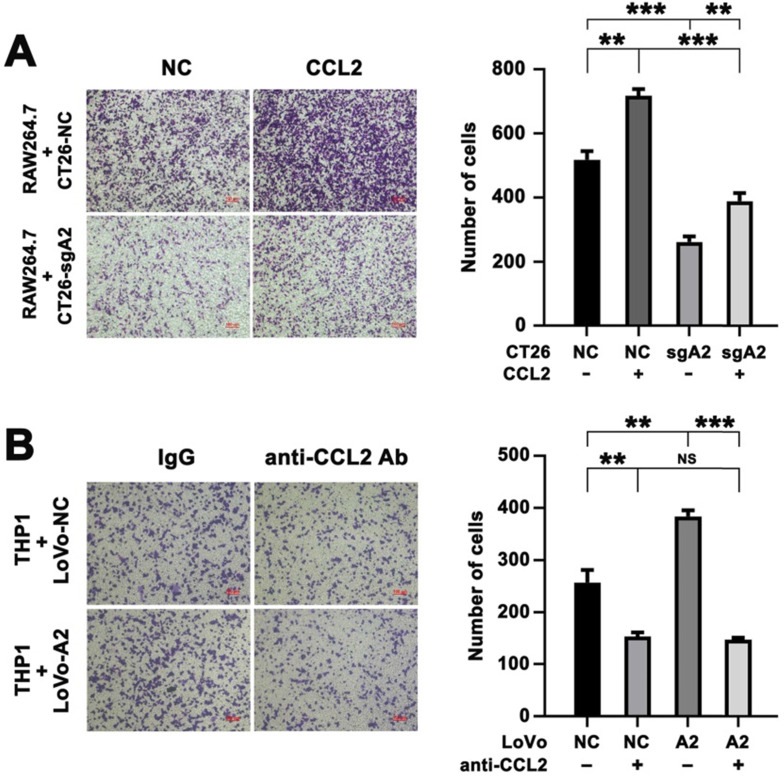
Increased migration of macrophages by HMGA2 overexpression in CRC cells depended on CCL2. A, Representative images (left panel) and quantification (right panel) of RAW264.7 cells co-cultured with indicated CT26 cells treated with control or recombinant CCL2 protein. B, Representative images (left panel) and quantification (right panel) of THP1 cells co-cultured with indicated LoVo cells treated with control IgG or anti-CCL2 antibody. Error bars indicate SD. **P < 0.01; ***P < 0.001; NS, not significant.

To investigate the role of CCL2 in the regulation of macrophage recruitment, CT26-NC and CT26-sgA2 cells were treated with recombinant murine CCL2 followed by co-culturing with RAW264.7 cells. Consistently, Hmga2 knockout repressed the migratory potential of RAW264.7 cells, whereas treatment with the recombinant CCL2 protein enhanced it (Figure 6A).

Furthermore, we preincubated LoVo cells (NC vs HMGA2) with a neutralizing anti-CCL2 antibody and then used the Transwell co-culture system to assess the migratory ability of THP1 cells. As illustrated in Figure 6B, our results demonstrated that overexpression of HMGA2 promoted migration of THP1 cells, whereas treatment with the anti-CCL2 antibody abrogated it, indicating that HMGA2 and CCL2 played crucial roles in macrophage recruitment. We also found that the induction of THP1 migration by co-culturing with LoVo-A2 was abrogated after neutralizing CCL2 (Figure 6B).

In addition, to explore whether Hmga2 facilitated TAM infiltration via a CCL2-dependent way *in vivo*, we conducted the rescue experiments in mice by using the neutralizing anti-CCL2 antibody. Mice were subcutaneously injected with CT26-sgA2+NC and CT26-sgA2+A2 cells followed by treatment with the control IgG or anti-CCL2 antibody. As expected, Hmga2 overexpression induced CD206 intensity, whereas anti-CCL2 antibody inhibited it. However, the induction of CD206 intensity by Hmga2 overexpression was abrogated by anti-CCL2 therapy *in vivo* (Figure S3B). Our results indicated that HMGA2 promoted TAM infiltration via a CCL2-dependent way *in vivo*. Together, these data indicated that HMGA2 overexpression promoted the migration of macrophages in a CCL2-dependent manner.

### Clinical significance of HMGA2 and CD68 expression in human CRC specimens

**Figure 7:**
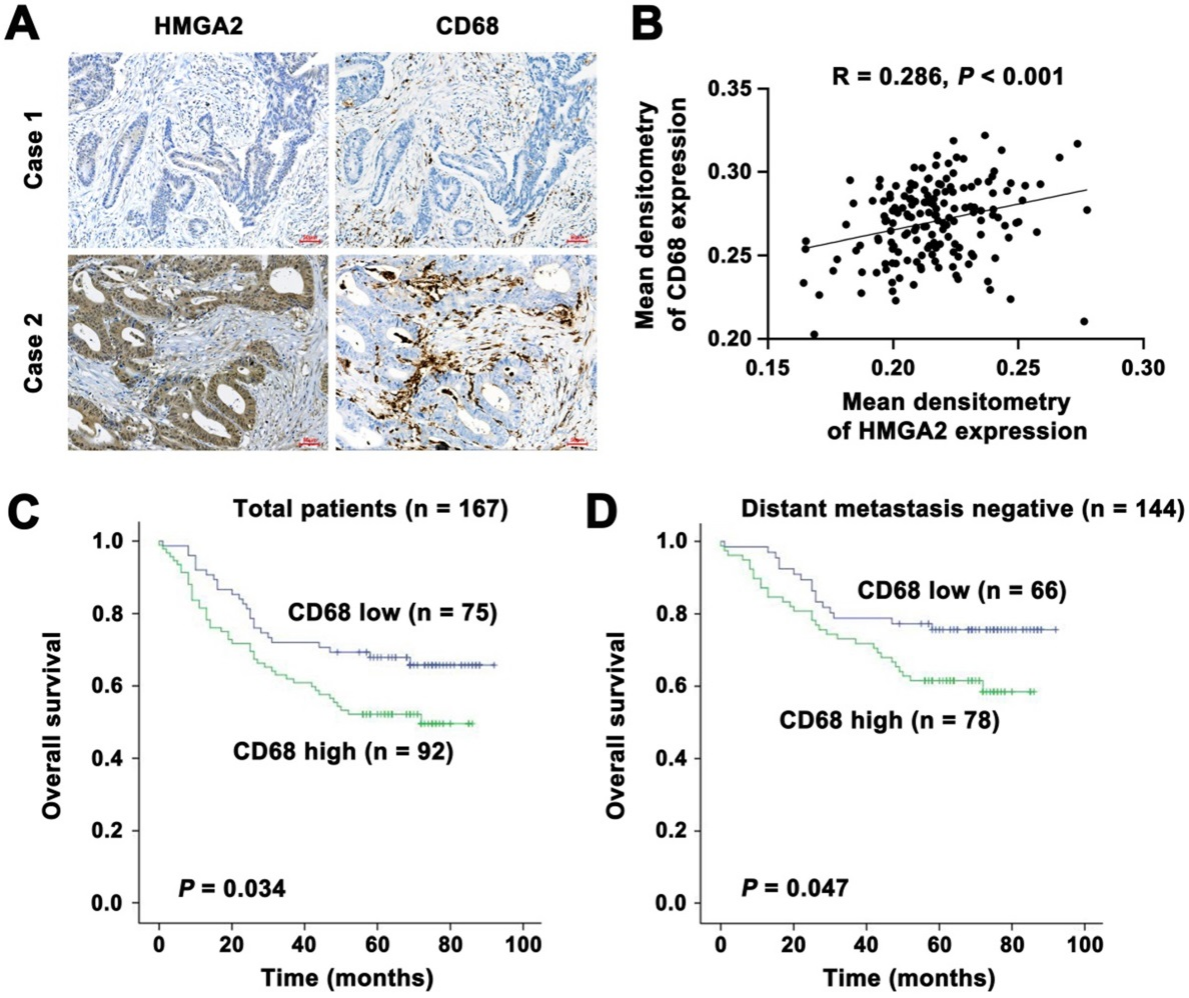
Clinical significance of HMGA2 and CD68 expression in human CRC specimens. A, Representative images of immunohistochemical staining of HMGA2 and CD68 in primary CRC tissues. Scale bar, 50 µm. B, Positive correlation between HMGA2 and CD68 expression. R = 0.286, *P* < 0.001. C-D, Kaplan-Meier survival curves for overall survival in all patients (C, n = 167) and in the subgroup with negative distant metastasis (D, n = 144) according to CD68 expression.

It has been reported that CD68 represents an immunohistochemical staining marker for macrophages 11. To investigate the association of HMGA2 levels with macrophage infiltration in CRC patients, we employed immunohistochemical staining to assess HMGA2 and CD68 expression in a panel of 167 human CRC specimens. Our results revealed a strong association between HMGA2 expression in tumor cells and CD68 expression in the stroma. As shown in Figure 7A-B, we found a trend of positive correlations between HMGA2 and CD68 expression (R = 0.286, *P* < 0.001). Next, we conducted the Kaplan-Meier survival analysis to evaluate the potential value of CD68 as a prognostic marker in CRC. As presented in Figure 7C, high CD68 expression in stroma correlated with reduced patient survival in all of the patients (*P* = 0.034). When stratified into distant metastasis positive and negative subgroups, we found that patients with high CD68 expressions had unfavorable overall survival, whereas patients with low CD68 expressions had favorable overall survival (Figure 7D, *P* = 0.047).

Then we analyzed data from the GEO database. As shown in Figure S4A-D, the level of HMGA2 expression was positively correlated with STAT3, CCL2 and TGFβ levels, whereas it was inversely associated with TNFα. These findings indicated that CD68 in the stroma could be used as a marker and predictor for clinical outcome, suggesting its clinical significance in CRC.

## Discussion

In recent years, immunotherapy has led to a great revolution due to its remarkable efficacy in the treatment of various cancers 16. However, immune cells with immunosuppressive properties in the TME limit its clinical benefit 17. Especially, the crosstalk between cancer cells and TAMs enables cancer cells to evade the immune defenses, thereby supporting cancer progression 18-20. As plastic cells, macrophages are classified into two distinct subsets. The pro-inflammatory M1 macrophages promote the immune response, whereas anti-inflammatory M2 macrophages facilitate tumor progression 21. They are induced by different surrounding conditions and differ in function, cytokine secretion, and signal transduction 22.

Emerging evidence has suggested the role of HMGA2 in TAM recruitment and polarization in cancer. Liu *et al.* illustrated that miR-340-5p inhibited macrophage recruitment by suppressing POSTN expression and decreased M2-TAM polarization in an LTBP-1-dependent manner. Furthermore, they also found that M2-TAMs reduced the expression of miR-340-5p by TGFβ-1-mediated HMGA2 expression in glioblastoma multiforme (GBM) 23. These findings highlighted the importance of a feedback loop between miR-340-5p and macrophages, where HMGA2 played a vital role in M2-TAM polarization in GBM. Consistently, immunohistochemical analysis showed that increased expression of HMGA2 positively correlated with TAM markers, including CD68, CD163, and CD204, in hepatocellular carcinoma (HCC) 24. Conversely, an opposite effect was observed from the study of M1 macrophages in the formation of cancer stem cells (CSC). Guo *et al.* found that HMGA2 enhanced M1-mediated CSC formation in breast cancer 25. It suggests that the effect of HMGA2 differs in different cancers. However, the network between HMGA2 and TAMs is poorly identified in CRC. To shed light on this issue, there is an urgent need to understand the effect and mechanism of HMGA2 in TAM recruitment and polarization. In the present study, we reported, to our knowledge for the first time, that HMGA2 overexpression in CRC cells led to increased TAM infiltration in the TME, thus creating an immunosuppressive environment.

STAT3 is a key oncogenic transcriptional factor that mediates signal transduction and regulates the transcription of target genes contributing to tumor development and progression 26. STAT3 activation is elicited by binding of cytokines (IL-6, IL-10, and IL-11) or growth factors (FGF and VEGF) to their corresponding receptors, leading to the recruitment and activation of Janus kinases (JAKs). Subsequently, JAKs phosphorylate cytoplasmic STAT3 protein on Tyr705 residue, resulting in the formation of STAT3 homo- or heterodimers, which are transported into the nucleus to regulate downstream gene transcription 15, 27. STAT3 was reported to participate in numerous biological processes in CRC development and progression, including cell growth 28, metastasis 29, stemness 30, apoptosis 31, angiogenesis 32, chemoresistance 33, and inflammation 34, 35.

As a key mediator of tumor-associated immune tolerance, STAT3 has been reported to be critical for the modulation of immune cells within TME in CRC, as outlined by many studies. Smith *et al.* found that STAT3 hyperactivation promoted MDSC accumulation and survival via upregulating DNMT1 and DNMT3b, to maintain an immunotolerant TME 36. The study by Wang and colleagues indicated that Th17 cells suppressed the infiltration of CD8^+^ T cells through IL-17A/STAT3/CXCR3 signaling in advanced-stage CRC 37. It was also reported that the JAK/STAT3 signaling exerts its immunosuppressive effects on FGFR2-mediated PD-L1 upregulation in CRC 38. In their study of the relationship between STAT3 expression and the density of immune infiltrate in the TME, Park and colleagues revealed that cytoplasmic STAT3 expression was negatively correlated with CD3^+^, CD8^+^ and FOXP3^+^ density by immunohistochemistry analysis in CRC 39. Furthermore, significant suppression of CAF and macrophage activation was observed after treatment with the inhibitor of IGF-1R and STAT3 in CRC 40. Consistently, Yeh *et al.* reported that IL-6 secreted by CRC cells promoted the phagocytic and migratory ability of macrophages 41. To our knowledge, we have, for the first time, reported that HMGA2 bound directly to the -743/-730 and -585/-576 promoter regions of *STAT3* and in turn promoted its transcription and expression, thus enhancing tumor immune evasion and facilitating tumor progression in CRC.

Chemokine (C-C motif) ligand 2 (CCL2), also known as monocyte chemoattractant protein-1 (MCP-1), is characterized by its chemoattractant activity for monocytes 14, 42. In TME, CCL2 secreted by tumor cells facilitates TAM recruitment and M2 polarization through the interaction with its receptor CCR2 43. TAMs, considered phenotypically similar to M2-like macrophages, contribute to cancer progression by producing anti-inflammatory and pro-tumorous cytokines 44. Several lines of evidence demonstrated that CCL2 was involved in regulating CRC tumorigenesis and progression in the TME. Chun and colleagues described that CCL2 enhanced polymorphonuclear (PMN)-MDSC activity in a STAT3-dependent manner, subsequently suppressing T cells and modulating CRC development 45. It was also reported that CCL2 facilitated liver metastasis of CRC by promoting CD11b/Gr1(mid) recruitment 46. Popivanova *et al.* stated that CCL2 increased macrophage infiltration and COX-2 expression, contributing to chronic inflammation-associated colon carcinogenesis 47. In addition, Kawada *et al.* found that CHI3L1 promoted IL-8 and MCP-1 secretion, eventually leading to increased macrophage infiltration in CRC 48. Interestingly, Grossman and colleagues revealed that the CCL2/CCR2 axis promoted TAM recruitment in liver metastasis, facilitating of CRC progression 49. Here, we reported that STAT3 increased CCL2 expression and in turn promoted TAM infiltration and M2 polarization in CRC.

Taken together, we found that HMGA2 in CRC cells induced TAM infiltration, M2 polarization and CCL2 production in TME of CRC *in vitro* and *in vivo*. Furthermore, our study revealed a novel mechanism, showing that HMGA2 directly promoted STAT3 transcription by binding to specific sequences in its promoter. Significantly, our results from human CRC specimens indicated that HMGA2 was positively correlated with CD68 expression, and the elevated CD68 expression in the stroma was significantly associated with poor prognosis in CRC. Our study further strengthened the value of HMGA2 in modulating TAM-mediated immune evasion in CRC. In summary, our present study demonstrated that overexpression of HMGA2 in CRC cells facilitated macrophage recruitment and M2 polarization via upregulating STAT3-mediated CCL2 secretion, thus promoting tumor immunosuppression in CRC. Our study revealed a novel pro-oncogenic effect of HMGA2 in the formation of an immunosuppressive microenvironment.

## Supplementary Material

Supplementary materials and methods, figures and tables.

## Abbreviations

TAMs: Tumor-associated macrophages
CRC: colorectal cancer
HMGA2: high-mobility gene group A2
AOM: azoxymethane
DSS: dextran sodium sulfate
ChIP: chromatin immunoprecipitation
TME: tumor microenvironment
Treg: regulatory T cells
Breg: regulatory B cells
MDSCs: myeloid-derived suppressor cells
TMAs: Tissue microarrays
ELISA: Enzyme-linked immunosorbent assay
RT-qPCR: quantitative reverse transcriptase-polymerase chain reaction
IHC: Immunohistochemistry
GBM: glioblastoma multiforme
HCC: hepatocellular carcinoma
CSC: cancer stem cells
STAT3: signal transducer and activator of transcription 3
JAKs: Janus kinases
CCL2: Chemokine (C-C motif) ligand 2
PMN: polymorphonuclear
