# Supplementary material for: HMGA2 facilitates colorectal cancer progression via STAT3-mediated tumor-associated macrophage recruitment
Source: Theranostics. 2022 Jan 1;12(2):963–75. doi: 10.7150/thno.65411 (PMC8692921; doi:10.7150/thno.65411)
Supplement: Supplementary file 1 — Supplementary materials and methods, figures and tables. [file thnov12p0963s1.pdf]

Supplementary Materials and Methods:

Cell culture and transfection:

Human CRC cell lines (LoVo and HT29), murine CRC cell lines (MC38 and CT26), human embryonic kidney cell line (HEK293T), human monocyte (THP1) and murine monocyte (RAW264.7) were obtained from American Type Culture Collection (ATCC, Manassas, USA). LoVo, HT29, CT26, THP1 and RAW264.7 were cultured in RPMI-1640 (Gibco) with 10% (v/v) fetal bovine serum (Invitrogen), while HEK293T and MC38 were cultured in Dulbecco′s Modified Eagle′s Medium with 10% (v/v) fetal bovine serum. For macrophage differentiation, THP1 cells were stimulated with 100 nM phorbol 12-myristate 13-acetate (PMA, Sigma, St Louis, USA) for 48 hours. Small interfering RNA (siRNA) targeting STAT3 (5’-CCACTTTGGTGTTTCATAATT-3’) or scramble control (5’-TTCTCCGAACGTGTCACGTTT-3’) were introduced into cells by using GenMute siRNA Transfection Reagent (SignaGen) according to the manufacturer’s protocol. To generate CRC cells that overexpressed HMGA2, we introduced lentivirus supernatants with control or HMGA2 overexpression constructs into CRC cells which were processed for stable selection by 2 mg/ml puromycin. Conversely, we employed short-hairpin RNA (shRNA) system to stably knockdown the expression of Hmga2 with target sequence (5’-GCAGTGACCAGTTATTCTT-3’) or scramble control (5’-ACTACCGTTGTTATAGGTGT-3’) in CRC cells. To generate knockout cells using CRISPR/Cas9 technology, CRC cells (MC38 and CT26) were transfected with lentivirus supernatants containing Cas9 and single guide RNA (sgRNA) targeting Hmga2 (5’-CACCTTCTGGGCTGCTTTAG-3’).

Generation of intestinal epithelial cell-specific Hmga2 knock-in (KI) mice:

Intestinal epithelial cell-specific Hmga2 KI mice were developed as described previously [1]. In brief, a fragment containing a CAG promoter, a loxP-neomycin-STOP-loxP cassette and Hmga2 CDS was inserted into the ROSA26 genomic locus. Then mice carrying the targeted allele were crossed with PVillin-Cre transgenic mice to obtain the intestinal epithelial cell-specific KI mice. All mice were

maintained in a specific pathogen-free facility in the Zhejiang University Laboratory Animal Center with the approval of the Ethics Committee of the Zhejiang University School of Medicine.

***In vivo* tumor xenograft model:**

Briefly, MC38 cells (1×10^6 in 100 μl PBS) were injected subcutaneously into C57BL/6 mice, while CT26 cells (1×10^6 in 100 μl PBS) were injected subcutaneously into BALB/c mice. The tumor sizes were measured every 5 or 3 days, and the tumor volume was calculated based on the formula: volume = (long dimension) $\times$ (short dimension)^2/2. Mice were humanely euthanized at the end of the analysis and all tumor samples were harvested for the following experiments. For *in vivo* treatment of Stattic, mice were received intraperitoneal injections of DMSO (5 mg/kg) or Stattic (MCE, 5 mg/kg) every 3 days starting on day 6. For *in vivo* treatment of the neutralizing anti-CCL2 antibody, mice were administered with a control IgG (BioLegend, 2 mg/kg) or neutralizing anti-CCL2 antibody (BioLegend, 2 mg/kg) every 3 days starting on day 6 by intraperitoneal injection.

Azoxymethane (AOM)/dextran sodium sulfate (DSS) mouse model:

Both C57BL/6 WT and intestinal epithelial-specific Hmga2 KI mice were subjected to the AOM/DSS model. As illustrated in Figure 2A, mice aged 8-10 weeks were administrated a single intraperitoneal injection of AOM (10 mg/kg body weight). Then they were fed by three cycles of 2.5% DSS in drinking water for 1 week followed by 2 weeks recovery. At week 10, small and large intestines were isolated and processed for further analysis.

***In vitro* co-culture assays:**

The *in vitro* co-culture assays were performed in the 0.4μm Transwell system (Corning). Macrophages or PMA-treated monocytes and CRC cells were seeded in the upper chambers and lower chambers of transwells, respectively. After 24 hours, the macrophages and CRC cells were harvested for the following qPCR analysis, while the medium was collected for macrophage migration assay with the 8μm Transwell (Corning). After culturing for 24 hours, the cells on the top surface of the

membrane were removed and the migrated cells on the bottom side were stained with 0.1% crystal violet. Then the number of cells was counted in three different randomly fields under a light microscope.

Isolation of leukocytes from tumor and intestinal tissue:

Tumor samples and intestinal tissue was isolated from *in vivo* tumor xenograft model and AOM/DSS mouse model, respectively. After trimming of necrotic tissues, the samples were washed twice with PBS and cut into 1-2 mm^3 pieces. Then they were digested in RPMI 1640 with 10% FBS, 1mg/ml collagenase IV and 20ug/ml hyaluronidase at 37℃ for 3-4h. The digested solution was filtered through cell strainer (40 μm), and then centrifuged at 300g for 5 min to collect single cells. Leukocytes were gained after gradient centrifugation from the interface between 40% and 80% percoll.

Flow cytometry:

Leukocytes were resuspended in pre-cooled PBS, gated by eFluor 450-labelled CD45 (1:40, eBioscience), and then incubated with indicated antibodies for 30 minutes at 4℃ in the dark, including APC-labelled CD11b (1:160, eBioscience), PE-labelled F4/80 (1:80, eBioscience) and PerCP/Cy5.5-labelled CD206 (1:40, Biolegend). Subsequently, the stained cells were centrifuged at 800 rpm for 5 minutes, and the supernatant was discarded. After washing three times with PBS, cells were resuspended in FACS buffer and then analyzed using a flow cytometer (Beckman Coulter, Brea, CA, USA).

Western blotting:

In brief, equal amounts of protein was resolved on SDS-PAGE and then transferred to Nitrocellulose membranes (EMD Millipore, Billerica, MA, USA). After blocking with 5% nonfat dry milk for 1 h at room temperature, the membranes were incubated with primary antibodies against HMGA2 (1:1000, Cell Signaling Technology), STAT3 (1:1000, Cell Signaling Technology), $pSTAT3^{Tyr705}$ (1:2000, Cell Signaling Technology) and β-actin (1:1,000, Cell Signaling Technology) for 24 h at 4℃. Subsequently, the membranes were incubated with secondary antibodies and analyzed

using Odyssey Infrared Imaging System (Li-COR, Lincoln, NE, USA). β-actin served as a loading control.

***In vitro* cultures of mouse intestinal explants and tumor samples:**

Tumor samples from *in vivo* tumor xenograft model and intestinal tissues from AOM/DSS mouse model were washed three times in pre-cooled PBS. They were cut into small pieces of 1.0 cm × 1.0 cm. Subsequently, each piece of tissues was cultured in 0.5 ml DMEM with 100 U/mL penicillin and 100 μg/mL streptomycin at 37°C for 24 h. Then, the supernatants were harvested and processed for ELISA.

Enzyme-linked immunosorbent assay (ELISA):

The concentrations of CCL2, TNF-α and TGF-β in the supernatants from cells and tissue cultures were quantitated by ELISA kits (R&D) according to the manufacturer's instruction.

Immunohistochemistry (IHC):

IHC assay was performed in TMA slides of 4 μm thickness. In brief, following deparaffinization, dehydration and antigen-retrieval procedures, TMA sections were blocked in 10% fetal bovine serum for 30 min at room temperature. We then used primary antibodies to incubate on sections overnight at 4 ℃, including anti-HMGA2 (1:50, Biocheck), anti-CD68 (1:200, BOSTER), anti-F4/80 (1:200, Cell Signaling Technology), and CD206 (1:200, BOSTER). The sections were then incubated with corresponding secondary antibodies and diaminobenzidine (DAB). Then, all photographs were captured and the Image-Pro Plus software (Version 6.0, Media Cybernetics, Rockville, MD, USA) was used to evaluate the immunostaining intensity of HMGA2 and CD68. They were calculated using the values of the integral optical density (IOD) and the area (AREA). And the average IOD/AREA of three fields reflect the protein expressions. For HMGA2, the score < 0.215 was considered as low expression, whereas score ≥ 0.215 as high expression. For CD68, the score < 0.270 was considered as low expression, whereas score ≥ 0.270 as high expression.

Public databases:

The CRC gene expression profiles were downloaded from GEO (https://www.ncbi.nlm.nih.gov/gds/). The mRNA expression data of HMGA2, STAT3, CCL2, TNFα and TGFβ were analyzed, and Pearson's R correlation coefficient was applied to determine the correlation between two groups. $P < 0.05$ was considered to be significant.

References:

1. Wang Y, Hu L, Wang J, Li X, Sahengbieke S, Wu J, et al. HMGA2 promotes intestinal tumorigenesis by facilitating MDM2-mediated ubiquitination and degradation of p53. J Pathol. 2018; 246: 508-518.

Supplementary Tables:

Table S1 Clinicopathologic characteristics in 167 CRC patients

Characteristic	Number (%)
Gender	
Male	94 (56.29%)
Female	73 (43.71%)
Age	
≤ 60 years	74 (44.31%)
> 60 years	93 (55.69%)
Tumor location	
Left-sided	46 (27.54%)
Right-sided	121 (72.46%)
Pathological type	
Tubular adenocarcinoma	123 (73.65%)
MC and SRCC	44 (26.35%)
T stage	
T1+T2	8 (4.79%)
T3+T4	159 (95.21%)
N stage	
Negative	89 (53.29%)
Positive	78 (46.71%)
M stage	
Negative	144 (86.23%)
Positive	23 (13.77%)
Clinical stage	
I+II	86 (51.50%)
III+IV	81 (48.50%)

MC, mucinous carcinoma
SRCC, signet-ring cell carcinoma

Table S2 Primers for RT-qPCR

Gene	Species	Sequences (5'-3')
CCL2	Human	F 5'-CAGCCAGATGCAATCAATGCC-3'
		R 5'-TGGAATCCTGAACCCACTTCT-3'
CCL2	Murine	F 5'-TAAAAACCTGGATCGGAACCAAA-3'
		R 5'-GCATTAGCTTCAGATTTACGGGT-3'
TNF-α	Human	F 5'-CCTCTCTCTAATCAGCCCTCTG-3'
		R 5'-GAGGACCTGGGAGTAGATGAG-3'
TNF-α	Murine	F 5'-CCTGTAGCCCACGTCGTAG-3'
		R 5'-GGGAGTAGACAAGGTACAACCC-3'

TGF-β	Human	F	5’-CTAATGGTGGAAACCCACAACG-3’
		R	5’-TATCGCCAGGAATTGTTGCTG-3’
TGF-β	Murine	F	5’-CTCCCGTGGCTTCTAGTGC-3’
		R	5’-GCCTTAGTTTGGACAGGATCTG-3’
IL-12b	Murine	F	5’-GTCCTCAGAAGCTAACCATCTCC-3’
		R	5’-CCAGAGCCTATGACTCCATGTC-3’
Stat3	Murine	F	5’-AGCTGGACACACGCTACCT-3’
		R	5’-AGGAATCGGCTATATTGCTGGT-3’
Hmga2	Murine	F	5’-GAGCCCTCTCCTAAGAGACCC-3’
		R	5’-TTGGCCGTTTTTCTCCAATGG-3’
GAPDH	Human/Murine	F	5’-ACCACAGTCCATGCCATCAC-3’
		R	5’-TCCACCACCCTGTTGCTGTA-3’

131 **Table S3 Primers for construction of STAT3 promoter in luciferase assays**

Gene	Amplification Site		Sequences (5’-3’)
STAT3	-1555/+133	F	5’-GCGTGCTAGCCCGGGCTCGAGTGCCCTGTAGATGCCTCTGTC-3’
		R	5’-CAGTACCGGAATGCCAAGCTTGGCCCCACCCTGCACCCC-3’
	-1555/-851	F	5’-GCGTGCTAGCCCGGGCTCGAGTGCCCTGTAGATGCCTCTGTC-3’
		R	5’-CAGTACCGGAATGCCAAGCTTGAAGACGGTGTATACGAAAGCTGA-3’
	-850 to -140	F	5’-GCGTGCTAGCCCGGGCTCGAGTGCATTCGCCTGTACGGG-3’
		R	5’-CAGTACCGGAATGCCAAGCTTCCCAGCCCCAGCCTGGCC-3’
	-1555/-140	F	5’-GCGTGCTAGCCCGGGCTCGAGTGCCCTGTAGATGCCTCTGTC-3’
		R	5’-CAGTACCGGAATGCCAAGCTTCCCAGCCCCAGCCTGGCC-3’
	-139 to +133	F	5’-GCGTGCTAGCCCGGGCTCGAGCGAGGATTGGCTGAAGGGG-3’
		R	5’-CAGTACCGGAATGCCAAGCTTGGCCCCACCCTGCACCCC-3’
	Mut 1	F	5’-AGTAGCCACTCTACGTCCACGTCATGTTTCCGGG-3’
		R	5’-GACGTAGAGTGGCTACTAGAGTGCGTGGGGAGAGG-3’
	Mut 2	F	5’-ATGTCCTCGGTGTCGTCCCCAGAACCCAATTCAGG-3’
		R	5’-GGACGACACCGAGGACATGGGTGACTCCGCCTG-3’
	Mut 3	F	5’-ACTCTCGCGCTAGCTGATTCCCGCGTGGTAAGA-3’
		R	5’-ATCAGCTAGCGCGAGAGTCCCCAGGTATCTCCAGATCC-3’
HMGA2	-1365/+140	F	5’-GCGTGCTAGCCCGGGCTCGAGGACATTTACACGCGCCTCCT-3’
		R	5’-CAGTACCGGAATGCCAAGCTTGAGCACAGGCAGAGGACAGAGT-3’

132 **Table S4 Primers for amplification of specific promoters in ChIP assays**

Gene	Amplification Site		Sequences (5’-3’)
STAT3	-1555/-1316	F	5’-TGCCCTGTAGATGCCTCTGTC-3’
		R	5’-CCTTCTCCCCAAGGATAGCTG-3’
	-1315/-1049	F	5’-AATGATGGGGGTAGGGAAAGA-3’
		R	5’-GAGTGATTTCTGTGTATGTGCTGATACT-3’

-1048/-816	F	5'-CATTATGCAAAGTTCATCCTCTATTATG-3'
	R	5'-ACCAGCTAGCCCATTGGCC-3'
-815 to -546	F	5'-CGGCGTTTGATGCTTGAAG-3'
	R	5'-TTGAGAGCCTCTTACCACGCG-3'
-551 to -350	F	5'-TCTCAACCTCGCCACCACG-3'
	R	5'-GCCACAGCGAGGGAAGAGC-3'
-349 to -159	F	5'-GGAGGGAGGAGCACCGAA-3'
	R	5'-AGGCACGCCGTCATGCAT-3'

Supplementary Figures:

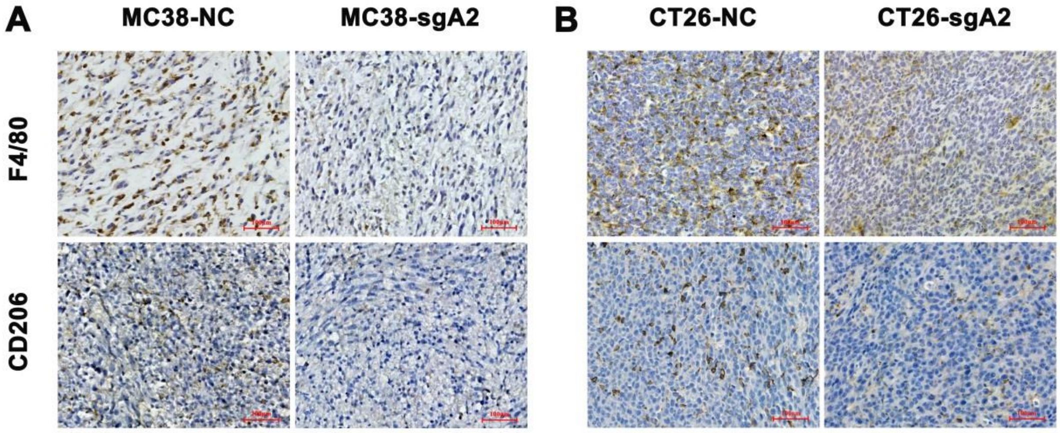

Figure S1 Knockout of Hmga2 in CRC cells suppressed TAM infiltration and M2 polarization in subcutaneous tumor models. A-B, IHC analysis of F4/80 and CD206 staining in tissues of MC38-NC/MC38-sgA2 xenograft tumors (A) and CT26-NC/CT26-sgA2 xenograft tumors (B).

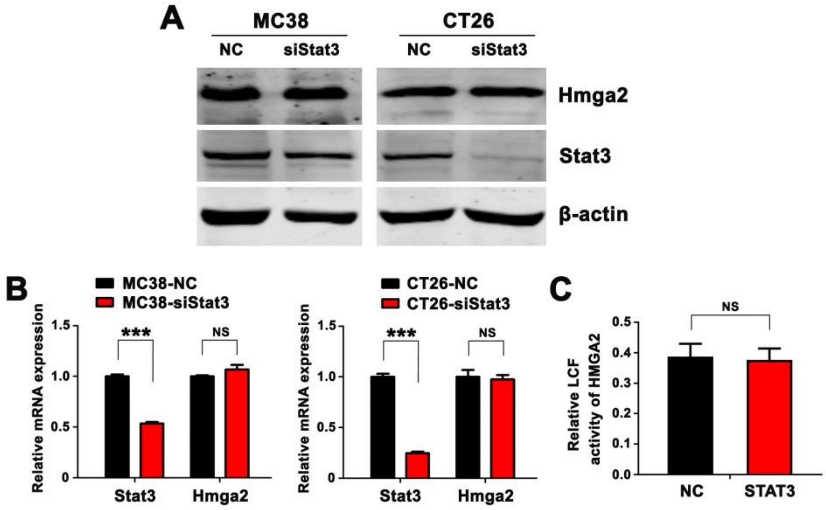

Figure S2 STAT3 did not regulate HMGA2 transcription. A-B, Western blot (A) and quantitative RT-PCR analysis (B) of Hmga2 and Stat3 in MC38 (left panel) or CT26 cells (right panel) transfected with control or siRNAs targeting Stat3. C, Luciferase activity of HMGA2 promoter constructs when co-transfected with control or STAT3-overexpressing plasmids in HEK293T cells. Error bars indicated SD.

***$P < 0.001$; NS, not significant.

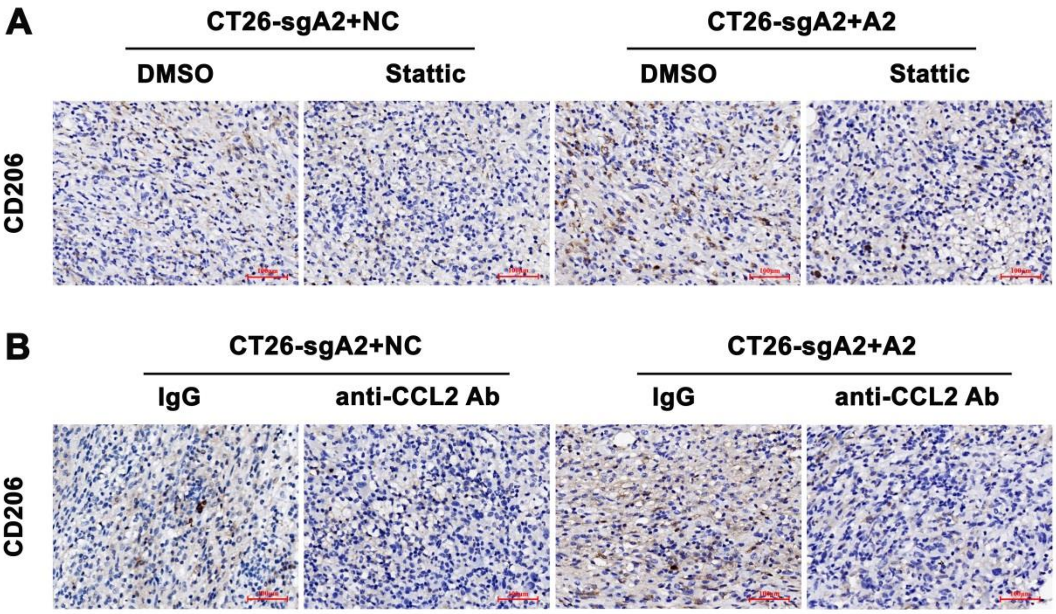

Figure S3 HMGA2 promoted TAM infiltration in a STAT3- and CCL2-dependent manner *in vivo*. A-B, Mice were subcutaneously injected by CT26-sgA2+NC and CT26-sgA2+A2 cells followed by the treatment of DMSO/Stattic (A) or IgG/neutralizing anti-CCL2 antibody (B). IHC analysis of CD206 staining were conducted in the indicated xenograft tumor tissues.

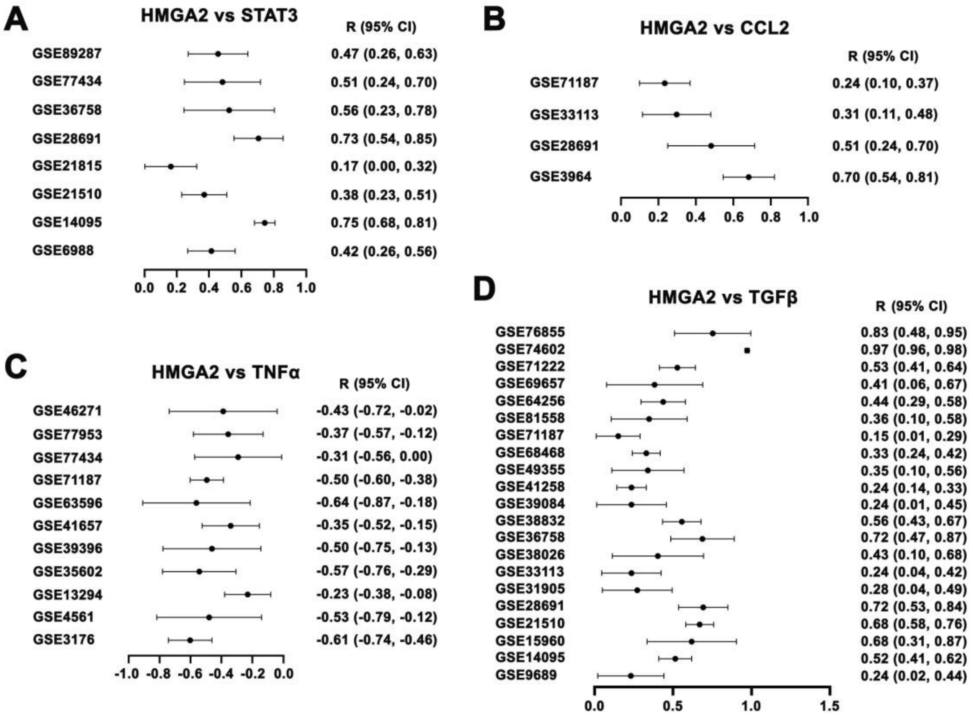

Figure S4 Correlation between the expressions of HMGA2 and STAT3, CCL2, TNFα or TGFβ. A-D, Forest plot showed that the level of HMGA2 expression was positively correlated with STAT3 (A), CCL2 (B) and TGFβ (D) levels, but it was inversely associated with TNFα (C) in GEO database. Correlation coefficient R was plotted with 95% confidence intervals (CI).
